# 

**Published:** 1890-08-02

**Authors:** 


### NOTICE TO CORRESPONDENTS.

All MS., Letters, Books for Review, and other matters intended for the Editor should be addressed The Editor, The Lodge, Porchester Square, London, W.

The Editor cannot undertake to return rejected M.S., even when accompanied by stamped directed envelope.

**Notice to Advertisers and Subscribers.**

All Advertisements, Orders for Copies of The Hospital, and Business Communications should be addressed to The Publisher, *not to the Editor*, The Hospital, 140, Strand, W.C.

**Bound Volumes of "The Hospital."**

Vol. VI., containing full page portraits of T.R.H. the Prince and Princess of Wales, and Miss Florence Nightingale, is obtainable at 4s., post free.

Orders will be received for Vol. VII., 4s. post free. Cases for binding may be had of the Publisher, at 1s. 6d. each.

**To Residents, Secretaries, and Matrons.**

The Editor will be glad to have the Regulations and each Report as issued by Asylums, Hospitals, Convalescent and Nursing Institutions, as well as those of other Charities, and an early copy in duplicate of all Appeals and Festival Notices, etc., addressed to *The Editor, The Lodge, Porchester Square, London, W.* Any superintendent, secretary, matron, or other chief official who regularly sends such information may be registered, on application to the Publisher, to receive free of cost a cover for binding The Hospital in half-yearly volumes.

### BURDETT'S HOSPITAL ANNUAL FOR 1890

Is now ready, and may be obtained of the Publisher, The Hospital Offices, 140, Strand, W.C., price 2s. 6d. limp boards, or 3s. 6d. cloth. All orders must be accompanied by a remittance.

### Books Received.

Balliere, Tindall, and Cox.

"Confidential Chats with Mothers." Mrs. Bowdich.

"The Book of Climates." D. H. Cullimore.

Bell and Sons, G.

"Physiology and Hygiene for Home Nursing." C. E. Fitzgerald, M.D.

Griffith, Farran, and Co.

"The Miracles of Healing." Rev. Dr. Belcher.

Lewis, H. K.

"Hygiene." Louis C. Parkes.

"Masso-Therapeutics." Murrell.

Simpkin, Marshall, and Co.
"Bench Book for Test Tube Work.' H. T. Lilley.

Thacker and Co.
"Indian Medical Service: Guide to Candidates and Officers." W. W. Webb.

### Scraps and Gleanings.

The fattest young lady in Paris is dead; she weighed 470 lbs., and was 19 years old.

The Royal Free Hospital makes a special appeal for £20,000 to re-build the west side of the hospital.

The Duke of Westminster has consented to become President of the Westminster Nursing Committee.

Mr. James Aveling, M.D., laid the foundation-stone of the Chelsea Convalescent Home at St. Leonards.

A concert was lately given at the galleries of the Jubilee Picture, Pall Mall, in aid of the Evelina Hospital.

Over two tons of bronze coin were received in the collecting-boxes of the metropolis on Hospital Saturday.

Messrs. Worthington and Ellgood, of Manchester, are to be the architects of the new Halifax Infirmary.

An International Congress on Cremation has been fixed to take place in Berlin from the 4th to the 6th of August.

The Trustees of Norfolk and Norwich Hospital have been presented with a portrait of Mr. Cadge, senior surgeon.

The *Leamington Courier* for July 5th contained a long account of the origin and progress of the Warneford Hospital.

Mr. Alderman Whitaker has resigned the post of secretary to Derby Infirmary, which he has held for fifty-one years.

The Alexandra Hospital at Woodhall Spa has removed to its own new building from its temporary home at Rose Cottage.

Miss Chippindale, of Tunbridge Wells, has sent a donation of £100 to the Royal Hospital for Diseases of the Chest, City Road.

Hertford will keep August 3rd as Hospital Sunday. Some of the inhabitants of the town have expressed their dislike to Sunday processions.

Mrs. Arabella Price, wife of a workman at Pengarnddu, has given birth to four children, which are living, but the poor woman herself died shortly afterwards.

The Raja of Venkatagiri has provided a new building for the Victoria Hospital at Ootacamund, and the Rajahs of Bobbili and Ramnaad have endowed new wards.

Lord Wolverton has consented to become a Vice-President of the London Skin Hospital, Cranbourn-street, and has sent a donation of ten guineas to that institution.

The Caxton Convalescent Home does not appeal to the general public for funds, but hopes for the support of men of literature and those connected in any way with the press.

Surgeon Parke, of the Emin Pasha Relief Expedition, has presented a collection of arrows, used by the dwarf tribes of Central Africa, to the Science and Art Museum of Dublin.

According to news from Iceland the epidemic of influenza has now spread all over the island. The complaint seems to be of a mild character, but several fatal cases have occurred.

It is reported that Mrs. Ayer, whose husband made a colossal fortune in the patent medicine business, proposes to erect a 3,000,000 dols. hospital for consumptives in New York.

The Dean of Windsor and Canon Duckworth lately attended a meeting in aid of St. Peter's Orphan and Convalescent Homes at Margate. The orphanage now contains 74 children.

The Aston Villa Football Club have been so generous towards the Birmingham Children's Hospital, they have been asked to nominate a representative for election as a Life Governor.

H.R.H. The Princess of Wales (the Patroness of the society) has this season sent a special donation of £25 to the Children's Country Holidays Fund (10, Buckingham Street, Strand).

It is proposed to establish a home for incurable ladies with very small means, in connection with the Convalescent Home at Hales, near Farnham, for which Miss Amy Mangles makes an earnest appeal.

The newly-appointed Leprosy Commission is to consist of three persons, a nominee of the Royal College of Surgeons, one of the Royal College of Physicians, and a member of the present Leprosy Committee.

The Queen has sent a donation of fifty pounds to the "Stanley Fund" now being raised at the Stanley and African Exhibition for placing a steamer on the Victoria Nyanza to advance civilization and Christianity in Africa.

The United Kingdom branch of the National Association for Supplying Female Medical Aid to the Women of India has granted a scholarship of £25 a-year for four years, tenable at the Edinburgh School of Medicine for Women.

The *Dublin Express* says the president and vice-president of the Royal College of Surgeons, Ireland, have been appointed to join a deputation from the licensing bodies to Mr. Stanhope on the subject of the grievances of the Army Medical Staff.

A collection has been made, for the second year in succession, in the London Commercial Sale Rooms, Mincing Lane, in aid of the Children's Country Holidays Fund (10, Buckingham Street, Strand). £70 10s. has been sent in to Mr. Alfred Lyttelton.

The bazaar recently held at Exeter Hall in aid of Band of Hope work throughout the United Kingdom has realised nearly £4,000. Towards this about £100 was contributed by the stall for the sale of Irish work presided over by Mrs. Lavens Ewart, of Belfast.

In order to make the Parkes Museum, which is supported by the Sanitary Institute, available to all classes for the purpose of obtaining information on matters relating to hygiene and sanitary appliances, the council have resolved to throw this museum open free at all times except when meetings or lectures are being held.

The foundation-stone of a new cottage hospital, which it is proposed to erect at Edington, near Bridgwater, in memory of the late Captain John Alexander Fownes Luttrell, was laid, with suitable ceremony, on July 3rd last by Miss Margaret Jane Fownes Luttrell, daughter of Mrs. Margaret Luttrell, at whose expense the institution is to be provided.

